# Perspectives on Abortion Services, the Pre‐Abortion Visit, and Telemedicine Abortion: A Qualitative Study in Sweden

**DOI:** 10.1111/psrh.12290

**Published:** 2025-01-12

**Authors:** Tagrid Jar‐Allah, Mina Edalat, Viola Nyman, Ian Milsom, Kristina Gemzell‐Danielsson, Johanna Rydelius, Helena Hognert

**Affiliations:** ^1^ Department of Obstetrics and Gynecology, Institute of Clinical Sciences, Sahlgrenska Academy University of Gothenburg, Sahlgrenska University Hospital Gothenburg Sweden; ^2^ Institute of Health and Care Sciences University of Gothenburg Gothenburg Sweden; ^3^ Department of Women's and Children's Health Karolinska Institutet, and WHO Collaborating Centre, Division of Gynecology and Reproductive Medicine, Karolinska University Hospital Stockholm Sweden

**Keywords:** abortion seekers, medical abortion, qualitative research, telemedicine, women's experiences

## Abstract

**Context:**

According to Swedish law, abortion treatment should be carried out at an approved healthcare facility. All persons seeking medication abortions are obliged to attend an in‐person visit, which includes a gynecological examination, an ultrasound scan, and administration of mifepristone at a hospital/clinic. However, some countries have implemented telemedicine abortion services without the requirement of in‐person visits during and after the COVID‐19 pandemic. The aim of this study is to (1) describe abortion seekers' experience of currently available abortion care; (2) explore abortion seekers' suggestions for improving medication abortion care; and (3) explore abortion seekers' views on an abortion care model that omits an in‐person appointment and examination.

**Methods:**

We interviewed 20 participants who sought early medication abortion at Sahlgrenska University Hospital, Gothenburg, Sweden, from March to April 2023. Systematic text‐condensation of qualitative semi‐structured interviews was used to explore abortion seekers' experience of the pre‐abortion visit, including the gynecological and ultrasound examinations, thoughts about abortion care services, and telemedicine abortion.

**Results:**

Three main findings emerged (1) The participants found it easy to contact the abortion clinic, but experienced undesired waiting time (2) Most participants appreciated the gynecological examination, but some found it distressing and uncomfortable (3) Participants considered telemedicine and taking mifepristone at home to be a good option in addition to in‐person abortion care.

**Conclusion:**

Offering both telemedicine and in‐person consultations enhances abortion seekers' autonomy, reduces delays, and minimizes stress in abortion care.

## 1 Introduction

Sweden's abortion law (from 1974) permits women to undergo an abortion, based upon their request, up to the 18th week of pregnancy. This right was extended to include non‐Swedish women from 2008. Abortion beyond 18 weeks requires approval from the National Board of Health and Welfare's legal council and after 21 weeks+6 days, abortions are not permitted if the fetus is considered viable [1]. Gynecological clinics manage these services, and they are not available at primary care facilities. In 2023 Sweden reported approximately 35,000 induced abortions, which is equivalent to a rate of 18 per 1000 women aged 15–44. Medication abortion is the most common abortion method used in Sweden, accounting for 97% of all abortions, which involves administering mifepristone followed by misoprostol 24 to 48 h later [2].

In‐person health care consultations, including those for abortion care, were restricted globally during the COVID‐19 pandemic to minimize the risk of contagion. This led to changes in clinical practice. In the UK, for example, the Royal College of Obstetricians and Gynaecologists (RCOG) adjusted the clinical guidelines for safe abortion in 2020 by allowing the first in‐person visit to be replaced by consultations via telephone or video call, and eliminating universal pre‐abortion testing requirements for medication abortions, often referred to as “no‐test medication abortions” [3, 4]. The estimation of gestational age was based on the date of the last menstrual period (LMP) while ultrasound scans were only performed if deemed medically indicated. By March 30, 2020, all authorities in Great Britain had issued emergency legal orders to permit use of mifepristone at home in combination with misoprostol [5]. In England and Wales, patients were eligible for no‐test medication abortions through telemedicine if their self‐reported LMP indicated a gestational age of less than 10 weeks and up to 12 weeks' gestation in Scotland [6, 7].

Several countries, implemented telemedicine models for abortion care, offering options such as telephone, web, or video counseling. These models allowed for remote counseling with mifepristone and misoprostol either mailed to patients or picked up from a clinic or pharmacy, eliminating the need for in‐person visits or physical examinations [8, 9, 10]. The telemedicine model has been found to be safe, effective, and person‐centered [11]. Several studies have also shown that most abortion seekers found self‐administrated early medication abortions supported by telemedicine to be very acceptable and a preferable option [12, 13]. Although one study reported that 13% of people still chose in‐person care [14]. The emergency regulation which was introduced in the UK during the COVID‐19 pandemic has been permanently retained, and this emergency measure introduced in 2020 is now part of standard practice.

In Sweden, transvaginal ultrasound examination is still the recognized gold standard of pre‐abortion assessment despite the international shift from in‐person consultation to the no‐test medication abortion protocol. Even if Sweden has a liberal abortion policy [15], the Swedish abortion law also mandates that abortion treatment has to be carried out at an approved healthcare facility. This means that abortion seekers must take the first abortion pill (mifepristone) at an inpatient‐clinic visit under the supervision of a healthcare professional. Since 2004, it has been permitted to let abortion seekers, in early pregnancy self‐administer the second abortion pills (misoprostol) at home, which is the choice of the majority [16]. According to these regulations, abortion seekers have to visit the clinic at least once for the first consultation, and sometimes twice if they cannot initiate the abortion with mifepristone during their first visit.

Currently, there is limited data on abortion seekers' experiences of abortion services and the pre‐abortion visit including the clinical examination and ultrasound scan in Sweden. A typical first consultation visit lasts for about an hour. It includes taking a medical history, performing an ultrasound scan, conducting a gynecological examination to screen for sexually transmitted infections (STI), and performing a bimanual examination.

There is also limited information about persons' opinions and acceptability of telemedicine abortion without an in‐person visit and self‐administration of mifepristone. However, the Swedish government has an ongoing legislative review on the abortion law, to study the possibility of starting and completing the full medication abortion at home [17], as is currently available in the UK and other countries.

In the present study, we aimed to (1) describe abortion seekers' experience of the current abortion care model which includes the in‐person consultation, the gynecological examination, and ultrasound scan, and to explore their opinion regarding access to abortion services and the associated waiting time; (2) explore abortion seekers' suggestions for improving medication abortion care; and (3) explore abortion seekers' views on an abortion care model that omits the in‐person appointment and examination. We believe this study will contribute valuable information that captures and reflects abortion seekers' perspectives on the entire abortion care process.

## 2 Methods

Qualitative semi‐structured interviews were conducted to explore persons' experience of the consultation visits before medication abortion and thoughts about abortion care services and in particular telemedicine abortion. Ethical approval was obtained from the Ethics Committee in Uppsala (registration number 2023‐00091‐01;February 15, 2023).

### 2.1 Participant Selection

We planned to interview 20 persons who sought early medication abortion at the abortion clinic located at the Department of Obstetrics and Gynecology, Sahlgrenska University Hospital (SU) in Gothenburg, Sweden. We deemed 20 interviews to be sufficient for data saturation, given the homogenous study population, and follow the principles of qualitative research methodology [18]. Persons aged 18 years or older who attended the abortion clinic for abortion counseling with a gestational age up to 10 weeks according to transvaginal ultrasound examination or the LMP, and understood and spoke Swedish or English were eligible to participate in the study. These abortion seekers intended to undergo a medication abortion with the administration of misoprostol at home or at the clinic. All potential participants received oral and written information about the study and participation was voluntary and anonymous without any economic compensation. Persons were invited to participate in the study either by telephone before the consultation visit or at the clinic before or after the consultation visit and ultrasound scan.

We selected the study site because, in the abortion clinic where the study was performed, the waiting room is shared by both women seeking abortions and those attending regular gynecological services. This creates a situation where abortion seekers may find themselves seated among other women accompanied by children or relatives of varying ages. We believed this setting could potentially influence the emotional experience of persons seeking abortion care and wanted to understand its impact.

### 2.2 Data Collection

We developed an interview guide with demographic questions and open‐ended questions to encourage the participants to talk about and explain their experiences about pre‐abortion visits and opinions about telemedicine. See the interview guide (Data S1). Further questions asked participants about taking mifepristone at the clinic or at home. Lastly, they were asked about how the abortion service in Sweden could be improved, and their thoughts about medication abortion without obtaining an examination. Pilot interviews were conducted with two participants to adjust and modify the interview guide.

The second author (MA) who works as a registered midwife at the abortion clinic and was not involved in the care of the abortion seekers, conducted 16 interviews after the first visit and four interviews when persons came back to complete the abortion at the clinic (after the administration of mifepristone). She conducted the interviews between March and April 2023 and the interviews lasted approximately 15–23 min each. All interviews were audio recorded and then professionally transcribed verbatim.

### 2.3 Data Analysis

The data were analyzed using systematic text‐condensation (STC) according to Malterud et al. [19], which is a descriptive and explorative method to describe the participant's experiences and opinions rather than exploring the meaning behind their answers. The STC consists of four steps. First, the first two authors (T.J., M.A.) read all the transcripts several times to establish an overview and get a general impression of the data to identify preliminary themes. All authors (T.J., M.A., V.N., I.M., K.G.D., and H.H.) read all interviews to attain a perspective on the analysis and to reduce single‐researcher preconceptions. After reading the transcripts, all authors discussed the preliminary themes. In the next step, the first and second authors reviewed the transcripts to develop codes and subcodes from the preliminary themes by identifying meaning units in the original text. All authors reached a mutual agreement about the content of the codes. In step three, the content was summarized and reduced into a condensate. Finally, (T.J., and M.A.) synthesized the condensed contents to generate generalized descriptions and concepts (recontextualized) and described the final themes in the presentation of results. The first author (T.J.) translated quotes from the transcripts into English, which the other authors then reviewed to confirm the correct translation and content validity. In reporting interviewees' perspectives and experiences, we assigned participant numbers to protect interviewee privacy.

## 3 Results

Forty potential participants were asked to participate in the study, twenty‐nine persons agreed to participate. Three persons declined to participate before the interviews. After the ultrasound scan, three participants had pregnancies with unknown location, two had gestational age of more than 10 weeks, and one participant wished to have a surgical abortion. Twenty participants were eligible to participate, of which five were interviewed before the consultation visit and ultrasound scan. At the time of the interview, eight participants had chosen medication abortion with home use of misoprostol, seven participants chose in‐clinic administration of misoprostol, and five had not yet decided the place for misoprostol administration. Participants' background and demographic variables are presented in Table 1. All participants gave written informed consent to be interviewed, and audio recorded. We present the findings in three main categories: before the first visit, the pre‐abortion visit and examination, and different abortion services options. For each of these, we describe the key themes identified from the interviews' analysis (Table 2).

**TABLE 1:** Participant demographics and clinical characteristics (*N* = 20).

Characteristic	*n* (%)
Age (y)	
Mean (SD)	28.9 (7.2)
Median (range)	26.5 (19–46)
Previous abortion	
Yes	6 (30)
No	14 (70)
Previous medical abortion	
Yes	5 (25)
No	15 (75)
Previous surgical abortion	
Yes	1 (5)
No	19 (95)
Use of contraception last 3 months	
Nothing	13 (65)
Oral contraceptive pills	4 (20)
Condom	3 (15)
Parity	
0	13 (65)
≥ 1	7 (35)
Education level	
Primary school	2 (10)
High school	4 (20)
Post high school	14 (70)
Place of birth, Sweden	
Yes	14 (70)
No	6 (30)
Waiting time for abortion visit (days)	
Mean (SD)	5.8 (2.3)
Median (range)	7 (1–10)
Waiting time for abortion decision (days)	
Mean (SD)	4,8 (7)
Median (range)	2 (0–25)
Previous knowledge about telemedicine abortion	
Yes	2 (10)
No	18 (90)
Employment status	
In paid employment	16 (80)
Student	2 (10)
Unemployed	2 (10)

*Note:* Data are presented as number (*n*) and percentages (%) for categorical variables. Continuous variables are presented with mean and standard deviation (SD), or median (range).

**TABLE 2:** Emerged themes.

Themes	
Experiences before the first visit	Ease of access Undesired waiting time Experiences in the clinic waiting room
Feelings about the pre‐abortion visit and examination	Perception of pre‐abortion gynecological examination Diversity of feelings about looking at the ultrasound screen
Opinions on different options of abortion services	Thoughts on taking mifepristone at a clinic or at home Telemedicine as an equivalent option to an in‐person visit

### 3.1 Experiences Before the First Visit

Three themes emerged: ease of access, undesired waiting time, and Experience in the clinic waiting room.

#### 3.1.1 Ease of Access

Most participants found it easy to contact the abortion clinic and obtain an appointment. The process was described as easy and efficient, reflecting the available comprehensive and accessible online information. This included clear contact phone numbers for the clinics and detailed and educational information about abortion services.

It was really easy. You just called, and then you got a time when you would be called back, and it was easy going to book my appointment, I had practically the whole week to choose from. So, it was very straightforward.—(Participant No. 1)

The online resources were described clearly and detailed, enabling abortion seekers to understand the procedures and what to expect, thereby reducing anxiety and uncertainty. The availability of multiple appointment slots within the week provided flexibility and convenience, accommodating various personal schedules, and making it simpler for persons to plan around their daily responsibilities. The efficiency of the process was a significant factor in the overall positive experience.

I found the information online really helpful. It was clear which number to call and also how everything would proceed. I found that everything was clear and well‐structured. so, I felt well‐informed before coming here.—(Participant No. 5)

#### 3.1.2 Undesired Waiting Time

Despite the initial ease of contact and appointment scheduling, some participants expressed dissatisfaction with the waiting period before their in‐clinic appointments.

It was easy to get in touch with the abortion clinic, and it was easy to get an appointment but there was a one‐week waiting period. I would have preferred an earlier appointment.—(Participant No. 3)

Participants described various physical and emotional experiences during the waiting period before the pre‐abortion visit. They reported physical symptoms such as nausea, vomiting, fatigue, bloating, and cramps, which made the waiting period more challenging. These symptoms were a constant reminder of pregnancy which contributed to emotional discomfort.

There was still some time, both good and bad, considering that I felt nauseous and, yes, hormones, and so on. I felt bloated and had cramps.—(Participant No. 8)

A few participants expressed concerns about the advancing gestational age during this time, which added pressure to their decision‐making process.

It was not good to wait, I was thinking that the baby is growing, and I thought that I hoped it wouldn't be a life before I … I had an abortion and if it did, I wasn't sure I could go through with it.—(Participant No. 12)

Only one participant described that she needed the waiting to be sure she had made the right decision about having an abortion.

I had thoughts about whether I could change my mind; if I could, it was quite a good amount of time I had to decide. I was also able to choose when I wanted to come, considering that I said the week after.—(Participant No. 8)

#### 3.1.3 Experiences in the Clinic Waiting Room

The waiting room played a separate role in shaping persons' experiences of abortion care. Most participants found waiting in the clinic waiting room acceptable and that it went quickly.

Waiting in the waiting room felt okay. The process was very quick, and I didn't have to wait long. I started filling out the health declaration form but had only managed to complete half of it before I was called in to meet the doctor.—(Participant No. 8)

One participant observed that sharing the waiting room with patients accompanied by babies might be uncomfortable for persons seeking an abortion, although she did not personally feel affected by this. Indeed, one participant expressed she felt like she was being judged and felt ashamed and avoided the waiting room.

I find it difficult. There are a lot of emotions, and it feels a bit like being judged, even though I understand that everyone might be there for the same reason, but it felt uncomfortable, and I stood a bit apart as well. I didn't sit down; I stood in the doorway so that no one could see me.—(Participant No. 6)

### 3.2 Feelings About the Pre‐Abortion Visit and Examination

Participants commented on how they experienced a friendly, educational, and informative reception, and that everyone at the clinic was supportive and pleasant. Many expressed that health professionals had a lot of sympathy and provided correct and professional care, which felt safe. Two themes emerged regarding the pre‐abortion visit: Perception of pre‐abortion gynecological/pelvic examination and a diversity of feelings about looking at the ultrasound screen.

#### 3.2.1 Perception of Pre‐Abortion Gynecological Examination

Most participants were willing to undergo the examinations to check gestational age and assess the pregnancy status. They experienced the doctor as skilled, and gentle, and experienced that they received sufficient information during the examination.

But it was good. No, it was very clear … it felt safe to be able to say when it hurt and where, like, it was on my terms.—(Participant No. 5)

However, for some participants, the experience of the gynecological examination, including the transvaginal ultrasound, was distressing. One participant described feelings of panic triggered by a traumatic history of childhood sexual abuse. Another participant found the examination because of an abortion to be difficult compared to when she was expecting her children.

I've always hated gynecological examinations. I find it really tough, and it's no exception now, and it's … I mean, when you've done it because of a desired pregnancy, it's still something beautiful, in a way, but I … I was a bit unsure about how I would feel.—(Participant No. 11)

#### 3.2.2 Diversity of Feelings About Looking at the Ultrasound Screen

Some participants chose not to look at the ultrasound screen during the examination because they considered that it would make the abortion more difficult. One participant found the visit itself to be good, but since she had children already, the ultrasound examination was tough when she saw the fetus on the screen.

I felt a bit curious, but still, it was better for me not to see it considering what it would have done. So, it was good that I didn't get to see it.—(Participant No. 8)

Another participant expressed that she was very certain about her decision to have an abortion, but she wanted to find out how advanced her pregnancy was. One participant appreciated not being asked if she wanted to look at the screen or not, because it made her feel that she could keep a distance from the pregnancy. However, some participants found it interesting and exciting to see, and one expressed that she wanted to look but was not able to because she was lying down.

It was very interesting and exciting to see the ultrasound screen (slight laughter).—(Participant No. 7)

### 3.3 Opinions on Different Options of Abortion Services

Two themes emerged: thoughts on taking mifepristone at a clinic or at home and telemedicine as an equivalent option to an in‐person visit.

#### 3.3.1 Thoughts on Taking Mifepristone at a Clinic or at Home

All participants received mifepristone in‐clinic, 14 participants received it directly at the pre‐abortion visit and six participants had to come back a few days later for mifepristone because they could not start the abortion treatment immediately since having the abortion at that time did not fit their schedule. The participants had varying opinions about the location where they preferred to take the first abortion pill, highlighting different needs and expectations. Some felt more comfortable taking it at the clinic as they believed that the presence of healthcare professionals offered a sense of safety in case they would experience any reactions or needed professional intervention.

Yes, but it still felt good. If you felt sick or vomited, you needed to get another pill within half an hour.—(Participant No. 9)

On the other hand, others favored the idea of taking mifepristone at home even if this option is not currently permitted. They found this option more convenient as it allowed them to fit it into their work or personal schedule and avoid visits to the clinic, which they found stressful. Furthermore, they believed that if they would have had the possibility to receive the pill in the mail and self‐administer it at home, it would have provided them with flexibility in terms of the timing of the procedure around their personal and professional commitments.

So, it would have been good with alternatives, that you could have chosen to do it all at home or come here. It's a bit easier to avoid coming back here to take the tablet, and you also preferably wouldn't want to do an ultrasound and gynecological examination.—(Participant No. 4)

#### 3.3.2 Telemedicine as an Equivalent Option to an In‐Person Visit

Overall, there was a range of feelings about telemedicine abortions. Some participants were very positive about telemedicine abortions for themselves or as an alternative method for women who need it. Others had mixed feelings; they were positive about telemedicine but preferred a thorough gynecological examination. A third group of participants were against telemedicine abortions due to safety reasons. The in‐clinic approach was valued for its comprehensive care, including gynecological and ultrasound examinations which many participants felt were crucial for their safety.

Actually, it does feel quite safe to have an examination, at least in my opinion. It's important to check how far along it is, you know. Of course, it depends on my menstrual cycle. This happened to me I should have been in my third period after childbirth and breastfeeding. So, it was still very irregular, you know. That's why I got pregnant because it was really strange. In this case, it feels good to have an examination. And then they also conducted tests for chlamydia and gonorrhea because it can lead to complications if you start bleeding. It can be very tough to have a gynecological examination, you know, but I'd rather double‐check everything that needs to be double‐checked than take the risk of not doing it. That's how I think, anyway.—(Participant No. 13)

On the other hand, participants expressed interest in telemedicine and viewed it as a beneficial alternative, especially for those who prefer avoiding the hospital environment or undergoing physical examinations, or find multiple visits to the clinic burdensome. Participants noted that telemedicine could spare abortion seekers the discomfort of waiting rooms and provide privacy, which is particularly valuable for those who face social or personal barriers, such as abusive relationships that restrict access to traditional care.

I think it's very good. I can't find … I can't imagine it's something that isn't good. It's free, it's confidential, it's accessible. So, I don't know, I have mixed thoughts about whether it would be easier or not. On one hand, yes, but it's good if you can get it with just one call, of course, the more accessible, the better for women.—(Participant No. 15)

Furthermore, participants discussed the potential for telemedicine to enhance accessibility for diverse groups, including persons from various cultural, and religious backgrounds, or those living in remote areas. They appreciated that telemedicine has the potential to minimize the waiting time and give the ability to self‐manage all parts of the abortion process, but concerns were raised about the risk for complications, such as undetected ectopic pregnancies.

Abortion shouldn't take too long; as soon as possible because not all people are the same. They come from different countries, cultures, and religions. Acting quickly makes it easier for women, and everything should happen as quickly as possible to support them. Telemedicine may help with this.—(Participant No. 16)

Most participants recognized the importance of having options for medication abortion, including the opportunity to choose between in‐clinic procedures and telemedicine services and the option to take mifepristone at home. The participants viewed these choices as important ways to enhance their autonomy and give them more control over their healthcare decisions.

well, I think the most important thing is that you can decide for yourself how you want to go through with your abortion. And that's already quite the case in Sweden, you can choose the method and whether you want to do it at a clinic or at home, depending on how early or late your pregnancy is. So, if this phone consultation option became another alternative, it could make it even more adaptable to each person's wants. That's really what I think … being able to decide for yourself how you want it. That's what's important.—(Participant No. 1)

## 4 Discussion

An in‐person pre‐abortion visit and administration of mifepristone at an approved clinic is still mandatory in Sweden, while many countries are now offering telemedicine options where no physical visit is required. In this study the participants were rather satisfied with the current abortion care services but some identified disadvantages of the current system could be addressed and solved by adding a telemedicine option alongside the in‐person standard care.

While most participants found it easy to contact the abortion clinic and schedule pre‐abortion appointments, some faced a challenging waiting period for their in‐clinic appointments. For many, waiting added unnecessary emotional and physical stress, particularly when combined with pregnancy symptoms. Studies have shown that women prioritize short waiting times and quality treatment over other factors such as the abortion method itself [20]. Studies, including Ralph et al., showed that most abortion seekers are highly certain of their decision, with uncertainty levels similar to or lower than other healthcare choices [20, 21, 22]. Other studies have found that waiting periods and two‐visit requirements do not significantly change decisional certainty for most people [23, 24, 25]. Telemedicine and home administration of mifepristone could potentially reduce or eliminate the waiting period, offering more timely care in line with WHO guidelines on quality healthcare services [26]. Only one participant expressed that she appreciated the waiting time and was afraid of the risk of making a rash decision. However, introducing telemedicine does not eliminate the possibility of a self‐chosen waiting period and offering emotional and psychological support when needed.

Many participants considered the gynecological examinations valuable, but those who found it distressing could have benefitted from a telemedicine procedure without a physical examination. As women in Sweden usually do not get routine gynecological examinations, many abortion‐seekers consider the pre‐abortion visit as an opportunity for general health control. However, some participants found these examinations distressing and uncomfortable, indicating that a telemedicine model without routine physical examinations could better meet their needs while maintaining safe and effective care.

Similarly, the experience of viewing the ultrasound screen varied. While some participants appreciated the information provided by ultrasounds, others found the experience emotionally distressing. Some studies have shown neither negative effects nor an influence on the woman's abortion decision [27, 28, 29]. while Blaylock et al. [21] revealed that while some women value the information gained from ultrasound, others find the process emotionally distressing regardless of if they view the ultrasound image or not. These findings highlight the need for personalized pre‐abortion care. Telemedicine models, which only ask for gynecological examination and ultrasonography when medically necessary, could reduce distress while ensuring safe and effective care.

The study suggests that introducing a telemedicine model could increase women's autonomy in abortion care services by offering the possibility to choose the kind of consultation they prefer. While most participants appreciated the in‐person contact they were offered they regarded telemedicine as a good complement and a crucial option for those who for various reasons cannot attend an in‐person consultation. Previous studies have shown that telemedicine is not only safe, efficient, and effective, but also a person‐centered method that provides women more flexibility and control over their healthcare decisions [11, 30]. In compliance with Swedish regulations, all participants had to take mifepristone at the clinic under the supervision of a healthcare provider. The participants had diverse views regarding their preferred location for taking the initial abortion pill. While some felt safer and had more support at the clinic, others appreciated the flexibility of taking the pill at home, highlighting the importance of offering abortion seekers choices based on their individual needs [31]. Allowing home administration of mifepristone could enhance autonomy while maintaining safety and efficacy, as demonstrated by a recent study by Danielsson et al. [7, 14, 32].

This study is limited by the self‐selected participants seeking abortion in Gothenburg, Sweden. We could only interview abortion seekers who came for an in‐person consultation and had mifepristone administered at the clinic. We could not include persons who had experienced a telemedicine abortion, since it is not yet allowed in Sweden. Furthermore, most participants were unfamiliar with telemedicine abortion before the interview, and they gave opinions about telemedicine based on the information they received during the interview. Despite this limitation, this is the first study to understand and capture abortion seekers' experiences regarding access to pre‐abortion appointments, gynecological and ultrasound examinations, and their opinions on telemedicine abortion in Sweden as a country with a liberal abortion policy [33]. There are no other studies, to our knowledge, that explored Swedish women's perspectives on the current abortion service options and particularly telemedicine.

## 5 Conclusion

This study shows that offering a telemedicine model will enhance abortion‐seekers' options by giving them greater choices in their healthcare decisions. A telemedicine model can reduce delays in accessing abortion care and eliminate the need for potentially stressful gynecological examinations for those who might have these experiences. The study also shows that offering the flexibility to take mifepristone at home rather than at a clinic can be convenient for persons choosing that option. Our study suggests the potential of offering care options tailored to individual needs. Telemedicine abortion could be one of these options.

## Conflicts of Interest

The authors declare no conflicts of interest.

## Supporting information


Data S1.
