# Supplementary material for: Perspectives on Abortion Services, the Pre‐Abortion Visit, and Telemedicine Abortion: A Qualitative Study in Sweden
Source: Perspect Sex Reprod Health. 2025 Jan 12;57(1):36–44. doi: 10.1111/psrh.12290 (PMC11936856; doi:10.1111/psrh.12290)
Supplement: Supplementary file 1 — Data S1. [file PSRH-57-36-s001.docx]

Supplementary document1: Interview guide

Is there anything you would like to ask before we start?

Patient Data:

1. How old are you?
2. Do you live alone or with family?
3. Where were you born? How many years have you lived in Sweden?
4. How many years have you studied? Highest achieved education level?
5. What is your main profession /occupation right now? (Working, Student, Job Seeking or other)
6. Have you been pregnant before?
7. Have you had an abortion before? How many times? Medical or surgical?
8. Do you have young children at home? If so, what age?
9. Have you used contraception in the last 3 months (before the abortion)? If so, which one?

• Women’s experience with access to abortion services.

1. How was it to seek/get an appointment at the abortion clinic?
2. How long did it take before you booked an abortion appointment after finding out you were pregnant?
3. How long did it take from your first phone contact with healthcare until you got an appointment for a gynecological examination at the abortion clinic?

• Women’s experience of the pre-abortion visits and ultrasound examination Initial Question: Can you start by freely talking about your feelings before the visit?

Support Questions:

1. How did it feel to wait for an appointment at the abortion clinic?
2. What were your thoughts and feelings?
3. How did it feel to wait in the waiting room?
4. What were your expectations before the visit?
5. How did you experience the visit with the doctor/midwife?
6. How did you experience the gynecological examination?
7. How did you experience the ultrasound examination?
8. Did you look at the ultrasound screen or not? How did you experience that?
9. How did you experience the information about contraception (contraceptive counseling)?
10. Have you chosen a contraceptive method you will use after the abortion? Which method?
11. Have you already received the Mifegyne pill, or will you pick it up later for the “home abortion”?
12. How do you experience the information on using the Cytotec pills and pain relief?

• Women’s thoughts on medical abortion in the future

1. What are your wishes for future abortion services in Sweden?
2. What can we do to improve the abortion service/process? in Sweden?
3. Have we missed considering anything important to you?

• Women’s thoughts on medical abortion without examination (Telemedicine) Initial Question:

- Have you heard about medical abortion without examination, so called telemedicine, being used in other countries?
- After the Covid-19 pandemic, telemedicine abortion services began to be offered in the UK, which means contacting the abortion clinic via phone or video call. Ultrasound examination is only done if indicated or at risk for abnormal pregnancy. The tablets can be picked up by the woman at the clinic or sent by mail.

What are your thoughts?

Support questions:

1. Do you want to undergo a gynecological and ultrasound examination?
2. Right now, the woman must take the Mifegyne pill in the clinic, what are your thoughts about that?
3. Would you prefer to take the tablet at home instead? Why?
4. What do you think about picking up the abortion tablets (Mifegyne, Cytotec, and pain relief) at the clinic without a gynecological/ultrasound examination?
5. Would you prefer to receive the tablets by mail instead? Why?

Closing Questions:

1. Do you have anything to add beyond what we have discussed?
2. What do you think is the most important of what we have talked about?
3. Thank you for participating. If you have any questions afterward, please feel free to contact me.
